# Phylogeny of Symbiotic Genes and the Symbiotic Properties of Rhizobia Specific to *Astragalus glycyphyllos* L.

**DOI:** 10.1371/journal.pone.0141504

**Published:** 2015-10-23

**Authors:** Sebastian Gnat, Wanda Małek, Ewa Oleńska, Sylwia Wdowiak-Wróbel, Michał Kalita, Barbara Łotocka, Magdalena Wójcik

**Affiliations:** 1 Department of Veterinary Microbiology, University of Life Sciences, 13 Akademicka st. 20–950 Lublin, Poland; 2 Department of Genetics and Microbiology, University of Maria Curie-Skłodowska, 19 Akademicka st., 20–033 Lublin, Poland; 3 Department of Genetics and Evolution, University of Białystok, 1J Ciołkowskiego st., 15–245 Białystok, Poland; 4 Department of Botany, Warsaw University of Life Sciences—SGGW, 159 Nowoursynowska st., 02–766 Warsaw, Poland; National Research Laboratory of Defense Proteins, REPUBLIC OF KOREA

## Abstract

The phylogeny of symbiotic genes of *Astragalus glycyphyllos* L. (liquorice milkvetch) nodule isolates was studied by comparative sequence analysis of *nodA*, *nodC*, *nodH* and *nifH* loci. In all these genes phylograms, liquorice milkvetch rhizobia (closely related to bacteria of three species, i.e. *Mesorhizobium amorphae*, *Mesorhizobium septentrionale* and *Mesorhizobium ciceri*) formed one clearly separate cluster suggesting the horizontal transfer of symbiotic genes from a single ancestor to the bacteria being studied. The high sequence similarity of the symbiotic genes of *A*. *glycyphyllos* rhizobia (99–100% in the case of *nodAC* and *nifH* genes, and 98–99% in the case of *nodH* one) points to the relatively recent (in evolutionary scale) lateral transfer of these genes. In the *nodACH* and *nifH* phylograms, *A*. *glycyphyllos* nodule isolates were grouped together with the genus *Mesorhizobium* species in one monophyletic clade, close to *M*. *ciceri*, *Mesorhizobium opportunistum* and *Mesorhizobium australicum* symbiovar *biserrulae* bacteria, which correlates with the close relationship of these rhizobia host plants. Plant tests revealed the narrow host range of *A*. *glycyphyllos* rhizobia. They formed effective symbiotic interactions with their native host (*A*. *glycyphyllos*) and *Amorpha fruticosa* but not with 11 other fabacean species. The nodules induced on *A*. *glycyphyllos* roots were indeterminate with apical, persistent meristem, an age gradient of nodule tissues and cortical vascular bundles. To reflect the symbiosis-adaptive phenotype of rhizobia, specific for *A*. *glycyphyllos*, we propose for these bacteria the new symbiovar “*glycyphyllae*”, based on *nodA* and *nodC* genes sequences.

## Introduction

Rhizobia are soil bacteria, capable of forming nitrogen-fixing symbiosis with fabacean plants, which is beneficial for agriculture and other environmental systems [1, 2]. The current taxonomy splits rhizobia into two groups, i.e.; “classical rhizobia”, affiliated to the genera; *Azorhizobium*, *Bradyrhizobium*, *Mesorhizobium*, *Rhizobium* and *Ensifer* (*Sinorhizobium*) as well as “new rhizobia”, comprising of bacterial species belonging to the genera; *Aminobacter*, *Devosia*, *Methylobacterium*, *Microvirga*, *Ochrobactrum*, *Phylobacterium*, *Shinella* (*Alpha-Proteobacteria*), *Burkholderia* and *Cupriavidus* (*Beta-Proteobacteria*) which for many years were not treated as fabacean symbionts [3]. Recently, in fabacean nodules additional endophytes were found, which were not able to induce nodules but entered into these structures together with rhizobia [3].

The taxonomy of rhizobia does not globally reflect the symbiotic features of bacteria, particularly their host plant range. It is well established that rhizobia with a broad host range are able to nodulate a high number of fabacean genera, as for example *Ensifer fredii* NGR234, which forms symbiotic interactions with 232 fabacean species, belonging to 112 genera and with non-fabacean plants of the genus *Parasponia* (the Cannabaceae family) whereas, rhizobia exhibiting a narrow host range can nodulate only a few hosts, e.g. *Rhizoium leguminosarum* bv. *trifolii* form nodules only on plants of the genus *Trifolium* [4, 5].

The effective rhizobium-fabacean symbiosis requires several classes of bacterial symbiotic genes, including *nif* ones encoding enzymes responsible for atmospheric nitrogen fixation and *nod* genes necessary for the synthesis of Nod factors, which act as nodule morphogenesis signals and trigger the plants towards nodule formation [6–8]. All Nod factors are β-1,4 linked N-acetyl-glucosamine oligomers ranging in length from 3 to 5 residues which are N-acylated at the non-reducing end and substituted by different chemical groups at both ends. The synthesis of the N-acylated chitin oligomers of the Nod factors is controlled by common *nodA*, *nodB* and *nodC* genes, that occur in all symbiotic rhizobia except for photosynthetic *Bradyrhizobium* genus strains BTAi1 and ORS278, which elicit root- and stem-nodules on *Aeschynomene sensitive* and *Aeschynomene indica* [9]. The common *nodABC* genes are spatially and functionally linked and they encode an acyltransferase, a chitin oligomer deacetylase and a chitin oligomer synthase, respectively [10–13]. The NodA and NodC proteins also determine the host range of the rhizobia. Various *nodA* gene products recognize and transfer specific fatty acids to the chitin oligomers whereas NodC proteins determine the length of the chitooligosaccharide chain. The other *nod* genes, e.g. *nodH*, *nodZ* and *nodS*, present in different combinations in rhizobial species, encode characteristic decorations of the Nod factors backbones and are primary determinants of host nodulation specificity [14–17].

The development of root nodules is a multistep-process, which drives rhizospheric rhizobia into an endocellular plant niche [18, 19]. In most cases, rhizobia are internalized in plant cells, *via* an endocytosis-like process, surrounded by a plant plasma membrane forming symbiosomes with bacteria (bacteroids) inside them and next, the fixation of atmospheric nitrogen can occur. Depending on the host genus, nodules induced by rhizobia can be “indeterminate”, with persistent meristematic growth or “determinate” characterized by an early cessation of meristematic activity and growth through the expansion of infected cells in the central part of nodule [19–21].

Presently, rhizobia within species are grouped into biovars (symbiovars) according to their symbiotic host-specificity and mainly on the basis of *nodC* gene phylogenetic analysis [3]. Jordan [22] was the first to use the term “biovar” to design three groups of *R*. *leguminosarum* bacteria, which are able to nodulate *Trifolium* sp. (*R*. *leguminosarum* bv. *trifolii*), *Vicia* sp. (*R*. *leguminosarum* bv. *viciae*) or *Phaseolus* sp. (*R*. *leguminosarum* bv. *phaseoli*). Later, these three symbiovars of *R*. *leguminosarum* were supported by the sequence analysis of *nodC* genes [23]. The concept of symbiovars seems to be correct in the case of plants restrictive for nodulation (e.g. *Trifolium* sp., *Vicia* sp., *Cicer* sp.) but this issue is still being discussed in the case of fabacean promiscuous for nodulation, that form symbiosis with different rhizobia biovars harboring different nodulation genes (e.g. *Phaseolus* sp.) [24, 25]. Currently, most symbiovars were determined using phylogenetic criteria based on the *nodC* [23, 26–29], *nodA* [29–31] and even *nifH* gene sequences [32–34] with a clear trend of using *nodC* gene to define symbiovars within rhizobium species. It seems, that in order to better understand rhizobium-fabacean symbiosis, the characterization and phylogenetic analysis of symbiotic genes should be included in minimal standards, when new fabacean symbionts are being described.

The aim of our study was determination of the phylogeny of the *nodACH* and *nifH* genes of *A*. *glycyphyllos* symbionts, affiliation of these rhizobia into symbiovar based on *nodA* and *nodC* gene sequence analyses, determination of the host plant range of studied rhizobia and description of the microscopic structure of *A*. *glycyphyllos* root nodules.

## Materials and Methods

### Bacterial strains and growth conditions

For the phylogenetic analysis of *nodACH* and *nifH* genes of *A*. *glycyphyllos* symbionts, the following six nodule isolates were used; AG1, AG7, AG15, AG27 (closely related to *M*. *ciceri* bacteria) and AG17 and AG22 (closely related to the bacteria of species *M*. *amorphae* and *M*. *septentrionale*) [35]. Furthermore, these strains and 22 other *A*. *glycyphyllos* nodulators described by Gnat et al. [35] were used in laboratory plant tests, in order to determine their host range and N_2_ fixation effectiveness. Additionally, in plant test *Mesorhizobium albiziae* CCBAU61158, *Mesorhizobium caraganae* CCBAU11299, *M*. *amorphae* ICMP15022, *Mesorhizobium chacoense* USDA4963, *M*. *ciceri* USDA3383, *Mesorhizobium gobiense* CCBAU83330, *Mesorhizobium huakuii* USDA4779, *Mesorhizobium loti* USDA3471, *Mesorhizobium plurifarium* USDA3707, *M*. *septentrionale* SDW018, *Mesorhizobium temperatum* LMG23931 and *Mesorhizobium tianshanense* USDA3592 were used in order to determine their ability to form symbiosis with *A*. *glycyphyllos*. The nodule bacteria were maintained at 4°C and cultured in a yeast extract-mannitol medium (YEM) at 28°C, as described by Vincent [36].

### Total DNA isolation

For the PCR-sequencing experiments, genomic DNAs were isolated from 30 ml of a 3-day-old bacterial culture in a YEM medium, according to the method of Pitcher et al. [37]. The purity and concentration of the DNA were determined with a spectrophotometer (Bio-Rad, Smart-Spec^TM^3000).

### PCR amplification of symbiotic genes and amplicons sequencing

The amplification reactions were performed with a ReadyMix^TM^Taq kit, following manufacturer’s specifications (Sigma). The 660-bp fragment of the *nodA* gene was amplified and sequenced using a forward primer, nodA-1 (5’-TGCRGTGGAARNTRNNCTGGGAAA-3’) and a reverse one, nodA-2 (5’-GGNCCGTCRTCRAAWGTCARGTA-3’) according to the procedure described by Haukka et al. [38]. The 890-bp long *nodC* gene fragment was amplified and sequenced with a forward primer, NodCFu (5’-AYGTHGTYGAYGACGGITC-3’) and a reverse primer, NodCI (5’-CGYGACAGCCANTCKCTATTG-3’) using the same cycling parameters as reported for this gene by Laguerre et al. [26]. The 567-bp fragment of the *nodH* gene was amplified and sequenced with forward, TSnodH1 (5’-VTKGAGYAACGGTGARYTGCTCA-3’) and reverse, TSnodH2 (5’-GCGAAGTGAWSCCGCAACTC-3’) primers, using the following conditions: preheating at 95°C for 2 min; 35 cycles of denaturing at 95°C for 45 s, annealing at 53°C for 30 s, and extension at 72°C for 2 min; and a final extension at 72°C for 7 min. A 780-bp long fragment of the *nifH* gene was amplified and sequenced with NifH1 (5’-CGTTTTACGGCAAGGGCGG-3’) and NifH2 (5’-TCCTCCAGCTCCTCCATGGT-3’) primers according to the protocol of Perret and Broughton [39]. PCR products were purified using Montage PCR Filter Units (Millipore, Massachusetts, USA) as recommended by the manufacturer. The purified amplicons were electrophoresed, in 1% agarose gel, to estimate the amount of DNA and then both strands of each amplified DNA were sequenced, with an ABI Prism BigDye Terminator Cycle sequence kit (Applied Biosystems, Foster City, CA, USA) and analyzed on a 3500 Genetic Analyzer (Life Technologies) sequencer, following manufacturer’s instructions. Sequences were deposited in the GenBank database under the accession numbers listed in the phylograms.

### Phylogenetic analysis

The symbiotic genes sequences of *A*. *glycyphyllos* nodule isolates and those of related bacteria from GenBank were aligned using ClustalW [40]. Next, they were manually corrected using GeneDoc software [41]. Phylograms were generated in MEGA program version 6.0 [42] using the neighbor-joining method (NJ) [43] based on Kimura’s two-parameter model (K2P) [44]. The stability of the strains groupings in the trees was estimated by bootstrap analysis (1,000 replicates) using the same MEGA software. The resulting trees were displayed using Tree View [45].

### Plant tests

All 28 *A*. *glycyphyllos* nodule isolates [35] were studied for their ability to form a symbiotic interaction with their native host (*A*. *glycyphyllos*), *Amorpha fruticosa*, *Astragalus cicer*, *Astragalus sinicus*, *Robinia pseudoacacia*, *Trifolium pratense*, *Lotus corniculatus*, *Medicago sativa*, *Ornithopus sativus*, *Phaseolus vulgaris*, *Glycine max*, *Vicia sativa* and *Lupinus luteus*. For plant tests, seeds were surface-sterilized [46], germinated on 0.8% agar-water plates and next, the obtained seedlings were placed in glass tubes filled with a N-free Hoagland's nutrient solidified with agar [47]. Three days later, the seedlings’ rootlets were inoculated with a rhizobial suspension (10^8^ cells per tube) and grown for six weeks at 21°C, with 14 h of light, per 24 h photoperiod. Non-inoculated plants, grown in a N-free Hoagland's medium [47], were included as negative controls. For each rhizobial strain-fabacean species, six replicates were prepared. The symbiotic properties of *A*. *glycyphyllos* rhizobia were evaluated by the presence and color of the nodules on inoculated plants, shoot dry weight and the acetylene reducing test, as an indicator of nitrogenase activity [48]. Additionally, the symbiotic interaction of *A*. *glycyphyllos* with; *M*. *albiziae* CCBAU61158, *M*. *caraganae* CCBAU11299, *M*. *amorphae* ICMP15022, *M*. *chacoense* USDA4963, *M*. *ciceri* USDA3383, *M*. *gobiense* CCBAU83330, *M*. *huakuii* USDA4779, *M*. *loti* USDA3471, *M*. *plurifarium* USDA3707, *M*. *septentrionale* SDW018, *M*. *temperatum* LMG23931 and *M*. *tianshanense* USDA3592 was studied in the plant test as described above.

### Statistical analysis of plant-test data

Date from plant tests, concerning symbiotic interaction of *A*. *glycyphyllos* and *A fruticosa* with rhizobium strains studied, were expressed as mean ± SD values. Differences between the studied groups of plants were analyzed, by a two-way analysis of variance (ANOVA) followed by Duncan’s multiple-range test (IBM SPSS Statistics 21, IBM Corporation, Somers, NY, USA). Differences at *p* < 0.05 were considered statistically significant.

### Light and electron microscopy

For microscopy studies, inoculated *A*. *glycyphyllos* plants (prepared as for the plant test) with the first nodules visible, were carefully pulled out of the glass tubes and transferred into pots, filled with a sterile perlite supplemented with a N-free Hoagland’s medium [47]. Plants were grown under 14 h light/10 h darkness photoperiod and whole root nodules were sampled 14 weeks after inoculation. The nodules were surface-cut for better penetration of reagents and fixed in a mixture of 5% glutaraldehyde and 4% paraformaldehyde in a 0.1 M Na-cacodylate buffer, pH 7.2, for 12 h under an air pressure of –0.06 MPa at room temperature. Samples were rinsed, contrasted and dehydrated, as described earlier [15]. Next, samples were embedded in a glycid ether 100 epoxy resin grade hard, which was prepared (and polymerized) according to the manufacturer’s formula (SERVA). Central longitudinal sections of 2 μm thick were cut in a sagittal plane and prepared for light microscopy, as described by Kalita et al. [15]. Ultra-thin sections, for ultrastructure observations, were placed on slot-grids coated with Formvar (SERVA), and treated as described by the same authors. Anatomical observations and micrographs were done using a light microscope Provis AX (Olympus). The ultrastructure was observed under a transmission electron microscope, Morgagni 268 (FEI Company) operating at 80 kV.

## Results and Discussion

Effective symbiosis between rhizobia and fabaceans, initiated by nitrogen starvation of the host plant, requires several bacterial symbiotic genes, including nitrogen-fixation (*nif*) ones, determining the reduction of N_2_ into ammonium and nodulation (*nod*) genes that encode Nod factors triggering nodule formation [7, 8, 49–51]. The basic structure of the Nod factors produced by different rhizobium species is very similar. It consists of a β-1,4-linked N-acetyl-D-glucosamine backbone, substituted at the non-reducing end by an acyl chain [13, 52]. Depending on the bacterial species, Nod factors can differ in the number of glucosamine residues present, the structure of the acyl chain and substitutions at the reducing and non-reducing terminal gluscosamine [11, 52, 53].

In this study, we have focused on the phylogeny of *A*. *glycyphyllos* symbionts’ common *nodA* and *nodC* genes, the *nodH* host specificity gene, as well as the *nifH* gene encoding dinitrogenase reductase. Phylograms of these four loci were compared, in order to determine whether their evolutionary history was similar and to explain whether *nod* and *nifH* loci were transferred across chromosomal lineages. The *A*. *glycyphyllos* nodulators studied represent different phenons and genomotypes of liquorice milkvetch symbionts [35, 54] and they, based on the 16S rRNA gene sequence analysis, were determined to be the close phylogenetic neighbors of *M*. *amorphae* and *M*. *septentrionale* (AG17 and AG22) and *M*. *ciceri* (AG1, AG7, AG15 and AG27) [35].

### The *nodA* gene phylogeny

**Fig 1:**
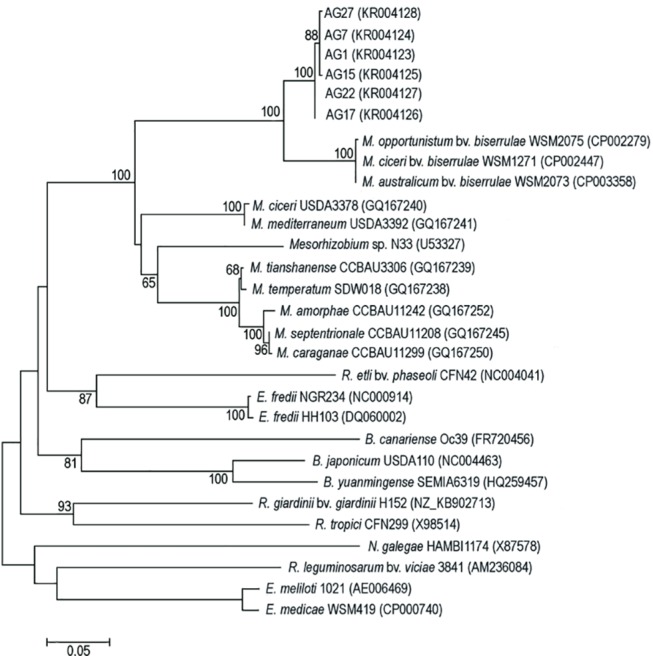
Maximum-likelihood phylogenetic tree based on partial *nodA* gene sequences of *A*. *glycyphyllos* symbionts (AG) and reference rhizobial strains. The GenBank accession number for each strain is shown in parentheses. Only bootstrap values of ≥ 60% (for 1,000 replicates) are indicated at the branching points. The scale bar indicates the number of nucleotide substitutions per site.

The nodulation gene *nodA* determines the type of N-acyl substitution on the nonreducing end of the Nod factor core and thus plays a significant role in determining the symbiotic specificity of nodule bacteria [8, 38, 53]. The 549 bp long, intragenic fragments of the *nodA* gene were sequenced for six *A*. *glycyphyllos* symbionts studied and aligned to *nodA* sequences of 24 reference rhizobia by CLUSTALW. The alignment showed 79 constant positions, 337 variable parsimony informative and 133 variable, but parsimony uninformative ones. The ratio of transitional to transversional pairs (ti/tv) was 0.94. In the phylogram of *nodA* sequences, liquorice milkvetch nodulators, with 99–100% *nodA* sequence identity to each other, were in the *Mesorhizobium* species cluster and formed a common group with them, at 86% bootstrap support (BS) (Fig 1). They were phylogenetically divergent from all other rhizobia included in the analysis and in the phylogram formed an independent branch with 100% BS. At the base of this lineage (as a separate cluster) *M*. *ciceri*, *M*. *opportunistum*, and *M*. *australicum* strains representing symbiovar *biserrulae*, with a 91–92% *nodA* sequence similarity to *A*. *glycyphyllos* rhizobia genes, were located. The *nodA* gene sequences similarities of *A*. *glycyphyllos* symbionts and other reference *Mesorhizobium* species, were in the range of 77–80%, and much lower to *nodA* sequences of the *Bradyrhizobium* sp., *Ensifer* sp., *Rhizobium* sp. and *Neorhizobium galegae*; i.e. 63–66, 66–69, 64–67 and 61%, respectively.

The most striking aspect of *nodA* gene evolution revealed by the *nodA* gene tree is the fact that all isolates from the *A*. *glycyphyllos* root nodules, regardless of their species designation, were recovered in one strongly supported clade, which indicates the possibility of lateral transfer of the *nodA* gene from a common ancestor to bacteria being studied. The 99–100% *nodA* sequence similarity of rhizobia specific for *A*. *glycyphyllos* points also to a relatively recent (in evolutionary scale) horizontal transfer of this gene to the bacteria being studied. The occurrence of horizontal transfer of the symbiosis genes was reported earlier by Sulivan et al. [55]. Due to this process, the mesorhizobia native to New Zealand, obtained *nod* genes (present in symbiosis islands) from an inoculant strain *M*. *loti* ICMP3153 and became able to N_2_ fixing association with the introduced plant, *L*. *corniculatus* [55–57]. A similar event took place in Australia, where local soil bacteria have acquired the ability to nodulate the pasture legume, *Biserrula pelecinus*, *via* lateral transfer to them of a symbiosis island from the commercial inoculant, *M*. *ciceri* bv. *biserrulae* strain WSM1497 [31]. Symbiosis genes of the genus *Mesorhizobium* bacteria are mobile, usually being found in plasmids or transmissible, chromosomal islands [16, 57–61].

### The *nodC* gene phylogeny

The *nodC* gene encoding N-acetylglucosaminyltransferase, responsible for the first step in Nod factor assembly, is related to the host range of the nodule bacteria [7, 12]. The 614 bp long, *nodC* genes alignment of milkvetch symbionts, studied here and 24 reference rhizobia, yielded information about *nodC* sequence similarities of these bacteria and showed 84 constant characters, 337 parsimony informative and 133 variable, but parsimony uninformative ones as well as revealed moderate bias towards transitions (ti/tv = 1.18).

**Fig 2:**
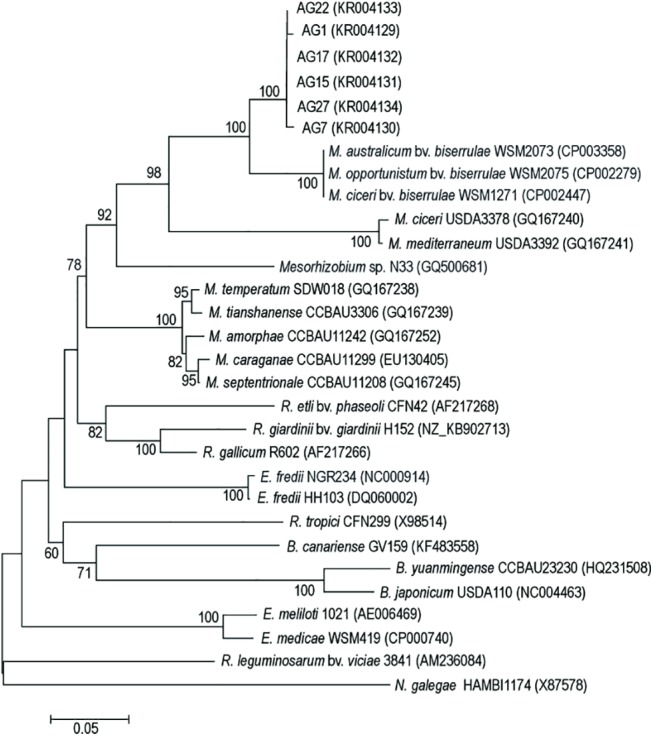
Maximum-likelihood phylogenetic tree based on partial *nodC* gene sequences of *A*. *glycyphyllos* symbionts (AG) and reference rhizobial strains. The GenBank accession number for each strain is shown in parentheses. Only bootstrap values of ≥ 60% (for 1,000 replicates) are indicated at the branching points. The scale bar indicates the number of nucleotide substitutions per site.

The analysis of the *nodC* sequences of *A*. *glycyphyllos* symbionts provided results congruent to those of the *nodA* genes. In the *nodC* gene phylogram (Fig 2), *A*. *glycyphyllos* nodule isolates, with a 99–100% sequence identity to one another, formed an independent branch close to a clearly separate cluster comprising of bacteria classified as symbiovar *biserrulae* i.e. *M*. *ciceri*, *M*. *opportunistum*, and *M*. *australicum* with a 92–93% sequence similarity to the rhizobia tested. The high similarity between the *nodC* sequences of liquorice milkvetch nodulators suggests that *nodC* genes of the bacteria studied have a monophyletic origin and derive from the same ancestor. As in the *nodA* gene dendrogram (Fig 1), the *Mesorhizobium* species and *A*. *glycyphyllos* symbionts (an 80–82% sequence similarity, not taking into account symbiovar *biserrulae* strains) formed a separate, well supported clade (78% BS). The *nodC* genes of the *Rhizobium etli* bv. *phaseoli*+*R*. *giardinii* bv. *giardinii*+ *R*. *gallicum*, *Ensifer fredii*, *R*. *tropici*, *Bradyrhizobium* sp., *E*. *meliloti+E*. *medicae* and *N*. *galegae*, with sequence similarities to *nodC* liquorice nodulators genes of 76–82, 77, 70–76, 74 and 68%, respectively, formed separate phylogenetic lineages on the *nodC* gene dendrogram. The grouping of *A*. *glycyphyllos* nodulators into one, clearly separate monophyletic cluster, close to the symbiovar *biserrulae* mesorhizobia and together with other *Mesorhizobium* species, shown in the *nodC* gene phylogram (Fig 2), was consistent with that presented in the *nodA* gene tree (Fig 1). Thus, both of these genes, of rhizobia specific for *A*. *glycyphyllos* nodulators, appear to share a common genealogy and probably derive from a single ancestor. It is highly probable that evolution of the host and changes in the host’s environment have exerted selection pressure on liquorice milkvetch symbionts and shaped the structure of the bacterial *nod* genes. It is also worth emphasizing, that *nodC* and *nodA* alleles of symbiovar *biserrulae* rhizobia split off at a basal position of the liquorice milkvetch symbionts, which correlates with the close relationship of these bacteria host plants; i.e. *Biserrula pelecinus* and *A*. *glycyphyllos* belonging to the same subtribe Astragalinae [62]. To reflect the genetically based symbiosis adaptive phenotype (ecotype), displayed by liquorice milkvetch nodulators, we propose the new symbiovar “*glycyphyllae*” for bacteria forming nitrogen-fixing associations with *A*. *glycyphyllos*. The *nodC* gene, encoding a chitin synthase and related to host specificity [7, 12, 26], is commonly used for symbiovar determination in rhizobia [28, 29, 31, 63–66]. Currently, within the genus *Mesorhizobum* three following symbiovars are described, i.e.: *biserrulae* (*M*. *ciceri*, *M*. *opportunistum* and *M*. *australicum—*symbionts of *B*. *pelecinus*), *loti* (*M*. *huakuii*, *M*. *loti* and *Mesorhizobium tarimense—*symbionts of *Lotus* sp.) and *ciceri* (*M*. *ciceri*, *Mesorhizobium mediterraneum*, *M*. *amorphae*, *M*. *tianshanense* and *Ensifer meliloti* **—**symbionts of *Cicer arietinum*). These symbiovars are clearly distinguished on the basis of *nodC* gene sequences and they illustrate the well adaptation of bacteria to their host plants [3, 29, 31, 63, 65, 66].

### The *nodH* gene phylogeny

**Fig 3:**
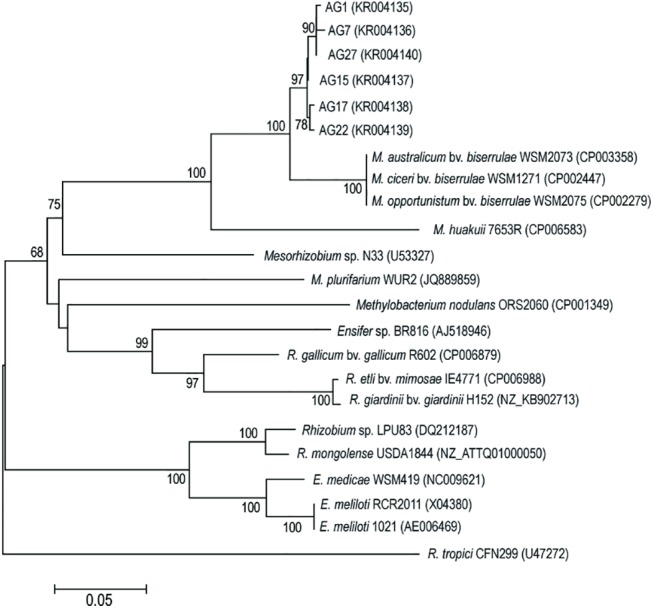
Maximum-likelihood phylogenetic tree based on partial *nodH* gene sequences of *A*. *glycyphyllos* symbionts (AG) and reference rhizobial strains. The GenBank accession number for each strain is shown in parentheses. Only bootstrap values of ≥ 60% (for 1,000 replicates) are indicated at the branching points. The scale bar indicates the number of nucleotide substitutions per site.

The *nodH* gene, which encodes sulfotransferase involved in the transfer of a sulfate group to the reducing end of the Nod factors [7, 14, 67–69], was identified in the *A*. *glycyphyllos* symbionts genomes by the PCR technique. The 491 bp fragment of the *nodH* gene was amplified in all six studied strains. Sequencing of the PCR products confirmed their similarity to the *nodH* genes of rhizobia, in which sulfated Nod factors were chemically supported [67–69]. The alignment of *nodH* gene sequences of liquorice milkvetch nodulators and reference rhizobia exhibited 180 constant positions, 80 variable but parsimony uninformative and 231 parsimony informative ones. The ti/tv ratio (1.11) points to a little bias towards transitions in the analyzed alignment. The obtained in this work *nodH* gene sequences (sharing 98–99% sequence identity with one another) were the most closely related to those of the *M*. *ciceri*, *M*. *opportunistum* and *M*. *australicum* sb. *biserrulae* strains (94% sequence similarity), and next, to the sequences of *M*. *huakuii*—symbiont of *A*. *sinicus* and *Mesorhizobium* sp. N33—symbiont of *Oxytropis arctobia* (subtribe Astragalinae) (83–84% and 76–77% sequence identity, respectively). Nucleotide identity of the *nodH* genes of *A*. *glycyphyllos* symbionts and other nodule bacteria, included in the analysis, was in the range from 67 to 74%. The phylogenetic analysis of the *nodH* sequences resulted in the NJ tree, presented in Fig 3. All six liquorice milkvetch nodulators were grouped within one, tight, monophyletic cluster (97% BS). In a sister, clearly independent clade, symbiovar *biserrulae* strains were placed (100% BS) and, at the base of this cluster, *M*. *huakuii* was located in a separate, highly supported branch. All these bacteria formed a strongly supported clade (100%) which indicates its robustness. Outside of this cluster, the other rhizobial strains harboring *nodH* genes were positioned. The presence of *nodH* genes in the genome of rhizobia specific for *A*. *glycyphyllos* suggests that these bacteria produce a 6-O sulfated Nod factor. Sulfation of Nod factors is an important determinant of host plant specificity and was documented in; *E*. *meliloti* (a symbiont of *Medicago* sp., *Melilotus* sp. and *Trigonella* sp.) [70], *R*. *tropici* (a symbiont of *P*. *vulgaris*, *Macroptilium atropurpureum* and *Leucaena leucocephala* [67], *Ensifer* sp. BR816 (a symbiont of *L*. *leucocephala* and *P*. *vulgaris*) [71], *M*. *huakuii* (a symbiont of *A*. *sinicus* [72], *Mesorhizobium* sp. N33 (a symbiont of *Astragalus* sp., *Onobrychis* sp. and *Oxytropis* sp.) [68] and *Methylobacter nodulans* ORS2060 (a symbiont of *Crotalaria podocarpa*) [73].

### The *nifH* gene phylogeny

**Fig 4:**
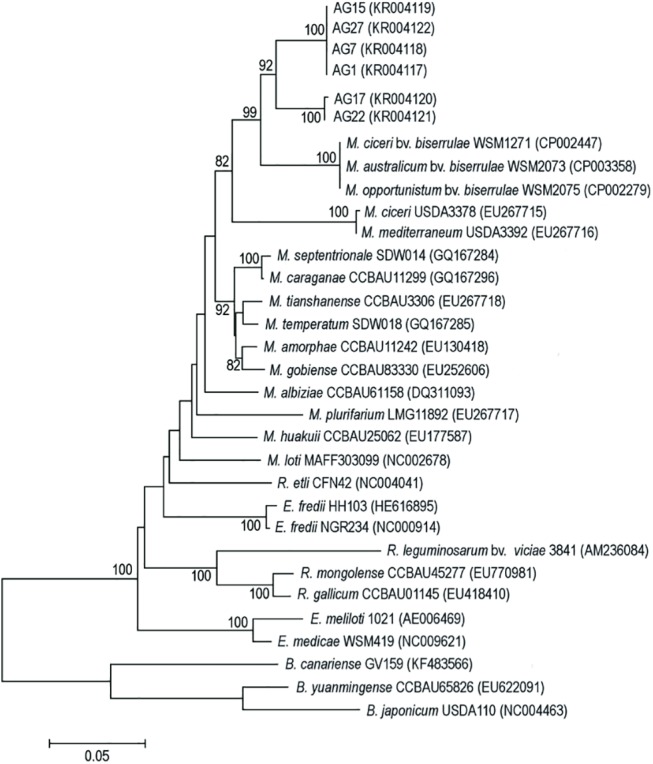
Maximum-likelihood phylogenetic tree based on partial *nifH* gene sequences of *A*. *glycyphyllos* symbionts (AG) and reference rhizobial strains. The GenBank accession number for each strain is shown in parentheses. Only bootstrap values of ≥ 60% (for 1,000 replicates) are indicated at the branching points. The scale bar indicates the number of nucleotide substitutions per site.

In our present study, on the phylogeny of symbiotic genes of *A*. *glycyphyllos* nodule isolates, the *nifH* gene encoding the Fe protein of the nitrogenase complex, was also sequenced and analyzed. The *nifH* gene sequences of strains studied and the reference rhizobia were aligned for a stretch of 602 nucleotides. This alignment exhibited 334 constant characters, 26 variable but parsimony uninformative and 242 parsimony informative ones. Additionally, a moderate transversion bias (ti/tv = 0.94) was also found. Bacterial strains isolated from *A*. *glycyphyllos* root nodules, with little variation across their *nifH* gene sequences (1%), were clustered together with the *Mesorhizobium* species (77–80% sequence similarity omitting symbiovar *biserrulae* strains) and formed a group clearly separate from all of them (Fig 4). Their closest phylogenetic neighbors were the *M*. *ciceri*, *M*. *opportunistum* and *M*. *australicum* sb. *biserrulae* strains which showed 91–92% *nifH* sequence identity with the studied milkvetch nodulators. The *A*. *glycyphyllos* symbionts formed two robust, closely related subclusters in the upper part of the *nifH* phylogram (AG17+AG22 and AG1+AG7+AG15+AG27) as in the 16S rDNA dendrogram [35]. In the branches, sister to those comprising of the mesorhizobia, the genus *Rhizobium* and *Ensifer* strains (sharing 64–69% identical sequences with bacteria tested) were placed. On the outskirts of the *nifH* gene tree, the *Bradyrhizobium* species, with 63–66% *nifH* sequence identity to *A*. *glycyphyllos* nodulators, were located (Fig 4). The *nifH* gene-based tree demonstrated a phylogenetic relationship of *A*. *glycyphyllos* symbionts, similar to that based on the common *nod* genes. Similar paths of the rhizobia *nifH* and common *nod* genes phylogenies may be explained by the close proximity of these genes to one another on symbiotic plasmids or chromosomal symbiotic islands, and by the lateral transfer of these mobile elements, mainly across species within genus and in some instances, between bacteria belonging to different genera [57–61]. The lateral transfer of symbiotic genes from a common ancestor, that lived in soil, to AG17 and AG22 strains (related to *M*. *amorphae* and *M*. *septentrionale*) as well as AG1, AG7, AG15 and AG27 isolates (related to *M*. *ciceri*) could enable these bacteria an effective nodulation of *A*. *glycyphyllos*. Generally, horizontal gene transfer plays an important role in the diversification and structuring of the natural population of rhizobia [31, 56].

### Host range and symbiotic efficiency

Rhizobia forming N_2_ fixing interactions with fabaceans exhibit various degree of symbiotic specificity, from very narrow as in the case of bacteria associated with plants of tribes; Trifolieae, Cicereae or Viceae [7] to broad host range (promiscuous) as for example strains isolated from nodules of *L*. *leucocephala*, *Gliricidia sepium*, *Prosopsis cineraria*, *P*. *vulgaris* or *Sesbania rostrata* [5, 7, 74, 75]. The host range and symbiotic effectiveness of 28 *A*. *glycyphyllos* root nodule isolates, classified as a moderately-slow growing mesorhizobia [35], were analyzed in laboratory plant tests on 13 fabacean species. All studied *A*. *glycyphyllos* mesorhizobia displayed narrow symbiotic efficiency in terms of the host range and N_2_-fixation capacity determined by shoot dry mass production and the acetylene reduction test (Table 1).

**Table 1:** Nitrogen-fixing efficiency of *A*. *glycyphyllos* nodule isolates in symbiosis with their native host plant and *A*. *fruticosa* as well as reference *Mesorhizobium* species in symbiotic interaction with *A*. *glycyphyllos*.

Rhizobium strains	Symbiosis effectiveness			
	Shoot dry weight (mg × plant^-1^) Mean ± SD^#^		Nitrogenase activity (nM ethylene × h^-1^ × plant^-1^) Mean ± SD^#^	
	*A*. *glycyphyllos*	*A*. *fruticosa*	*A*. *glycyphyllos*	*A*. *fruticosa*
AG1	11.59±1.62^a^	6.22±1.31(Nod^-^)^a*^	227.68±45.26^a^	NT (Nod^-^)
AG2	13.00±1.24^a^	12.00±2.22^b^	209.82±29.60^a^	99.23±25.67^a*^
AG3	11.75±0.98^a^	11.43±1.96^b^	156.87±50.11^a^	69.42±11.92^a*^
AG4	14.00±1.76^a^	12.00±1.91^b^	227.68±52.22^a^	125.48±18.97^b*^
AG5	12.25±1.12^a^	6.51±0.96(Nod^-^)^a*^	154.43±49.86^a^	NT (Nod^-^)
AG6	14.25±1.25^a^	12.00±1.85^b^	398.43±89.32^b^	80.39±16.26^a*^
AG7	11.75±0.73^a^	11.99±1.14^b^	420.60±91.50^b^	77.78±18.65^a*^
AG8	16.25±1.58^b^	11.92±1.66^b*^	473.21±97.87^b^	102.66±18.67^a*^
AG9	14.25±1.54^a^	11.67±1.89^b^	320.68±65.55^b^	85.54±20.49^a*^
AG10	11.75±0.81^a^	5.95±0.88(Nod^-^)^a*^	321.54±72.12^b^	NT (Nod^-^)
AG11	12.75±1.77^a^	6.21±1.77(Nod^-^)^a*^	322.43±85.66^b^	NT (Nod^-^)
AG12	13.51±1.87^a^	11.95±1.21^b^	256.78±65.18^a^	100.90±12.68^a*^
AG13	12.86±1.50^a^	6.11±1.05(Nod^-^)^a*^	223.21±38.16^a^	NT (Nod^-^)
AG14	11.75±0.56^a^	5.98±1.94(Nod^-^)^a*^	350.71±77.63^b^	NT (Nod^-^)
AG15	10.75±0.77^a^	11.53±1.22^b^	409.98±75.35^b^	76.56±12.91^a*^
AG16	11.57±0.39^a^	6.51±1.34(Nod^-^)^a*^	167.36±19.11^a^	NT (Nod^-^)
AG17	14.00±2.02^a^	7.00±1.22^a*^	151.79±22.62^a^	70.36±15.66^a*^
AG18	11.00±0.45^a^	6.62±1.65(Nod^-^)^a*^	212.94±26.98^a^	NT (Nod^-^)
AG19	13.75±1.82^a^	11.99±2.03^b^	210.21±19.35^a^	101.90±11.98^a*^
AG20	8.00±2.12^c^	5.89±0.99(Nod^-^)^a^	187.54±20.00^a^	NT (Nod^-^)
AG21	11.50±0.59^a^	11.88±1.13^b^	245.67±84.24^a^	81.84±17.79^a*^
AG22	13.25±1.43^a^	6.54±1.12(Nod^-^)^a*^	287.65±22.12^b^	NT (Nod^-^)
AG24	13.50±1.39^a^	5.84±0.56(Nod^-^)^a*^	299.11±74.21^b^	NT (Nod^-^)
AG25	12.00±1.31^a^	11.73±1.25^b^	304.65±86.66^b^	82.50±12.53^a*^
AG26	13.75±2.14^a^	11.67±2.05^b^	345.76±20.25^b^	88.39±13.66^a*^
AG27	13.50±1.96^a^	11.91±1.67^b^	290.18±35.50^b^	97.78±18.21^a*^
AG28	15.00±2.90^a^	6.57±1.45(Nod^-^)^a*^	245.78±60.34^a^	NT (Nod^-^)
AG29	11.58±0.14^a^	6.23±1.98(Nod^-^)^a*^	212.94±30.32^a^	NT (Nod^-^)
*M*. *caraganae*CCBAU11299	12.30±0.66^a^	NT	181.00±11.50^a^	NT
*M*. *ciceri*USDA3383	12.10±1.21^a^	NT	198.00±18.61^a^	NT
*M*. *loti*USDA3471	16.00±0.91^b^	NT	166.00±16.00^a^	NT
*M*. *septentrionale*SDW018	10.12±0.62^a^	NT	156.00±15.56^a^	NT
Uninoculated control	7.08±1.13^c^	6.34±1.52^a^	NT	NT
Source of variation: ANOVA (*p*—values)				
strain	0.0000		0.0000	
host plant	0.0000		0.0000	
strain × host plant	0.0000		0.0000	

SD, standard deviation; Nod^-^, nodules were not formed; NT, not tested (uninoculated plants and non-nodulated plants were not checked for acetylene reduction).

^#^Values represent the mean ± SD of six replicates. Means in the same column marked with different superscript letters (a, b, c) and means in the same line (separately for the shoot dry weight of plants and nitrogenase activity of rhizobia) marked with an asterisk (*) are significantly different (*p*<0.05); *p* values were calculated using the two-way ANOVA model followed by Duncan’s multiple-range test.

The liquorice milkvetch symbionts studied here formed pink, cylindrical nodules with substantial nitrogenase activity when inoculated on their original fabacean host, i.e. *A*. *glycyphyllos*. 54% of these bacteria also developed nodules on *A*. *fruticosa* but they were substantially less efficient in N_2_ fixation with this host than with *A*. *glycyphyllos*. Six weeks after inoculation of *A*. *glycyphyllos* and *A*. *fruticosa* with the test bacteria, the shoot dry weight of plants with root nodules was significantly different (p<0.05) from that of–N controls and ranged from 8.0 to 16.25 mg (uninoculated control from 5.96 to 8.21 mg) and from 7.0 to 12.0 mg (uninoculated control from 4.82 to 7.86 mg) per plant. Furthermore, the acetylene reduction activity of rhizobial symbionts, used as an indicator of nitrogenase activity (N_2_ fixation capacity), was significantly higher in inoculated *A*. *glycyphyllos* than in inoculated *A*. *fruticosa* and achieved the values of 151.79–473.21 and 69.42–125.48 nM per hour, per single plant, respectively. None of the *A*. *glycyphyllos* isolates nodulated; *A*. *cicer*, *A*. *sinicus*, *R*. *pseudoacacia*, *T*. *pratense*, *L*. *corniculatus*, *M*. *sativa*, *O*. *sativus*, *P*. *vulgaris*, *G*. *max*, *V*. *sativa* and *L*. *luteus*. It is notable that host range is one of the critical criteria in the identification and characterization of rhizobia and plant inoculation tests, with selected hosts, are required for the description of novel rhizobial strains [76].

Fabaceans, similar as rhizobia, exhibit also a different range of symbiotic specificity and single plant species may associate with several rhizobial genomotypes as for example *R*. *pseudoacacia* [7] or only with unique root-nodule bacteria, e.g. *B*. *pelecinus*, a pasture species [77]. In laboratory plant test, *A*. *glycyphyllos* was effectively nodulated by; *M*. *caraganae*, *M*. *ciceri*, *M*. *loti* and *M*. *septentrionale* but not by *M*. *albiziae*, *M*. *amorphae*, *M*. *chacoense*, *M*. *gobiense*, *M*. *huakuii*, *M*. *plurifarium*, *M*. *temperatum* or *M*. *tianshanense*. The dry weight of green part of nodulated *A*. *glycyphyllos* plants, six weeks after inoculation, was significantly higher than that of uninoculated control plants showing that *M*. *caraganae*, *M*. *ciceri*, *M*. *lot* and *M*. *septentrionale* fix atmospheric nitrogen and supply it to the symbiotic partner (Table 1). This conclusion was supported by acetylene reduction test (an index of nitrogenase activity) (Table 1), which showed that nitrogenase activity of above-mentioned *Mesorhizobium* species in symbiosis with *A*. *glycyphyllos* is lower than that of studied liquorice milkvetch nodule isolates (Table 1). The *Astragalus* species promiscuity was described earlier and it is known, that these fabaceans are nodulated by *Mesorhizobium*, *Rhizobium*, *Sinorhizobium* and *Bradyrhizobium* species, harboring diverse symbiotic genes, mainly by the genus *Mesorhizobium* strains [1, 78]. The mechanisms of *Astragalus* sp.-rhizobium promiscuity is not explained. It can be associated with a variety of host-released flavonoids, i.e. rhizobial *nod* genes inducers and with variety of Nod factors released by microsymbionts [7, 79].

### The microscopic structure of *A*. *glycyphyllos* root nodules

Symbiotic interaction between diazotrophic rhizobia and fabaceans leads to formation of root and occasionally stem nodules classified into two major types, i.e. determinate and indeterminate based on their morphology, development, and physiology. The nodule structure is largely determined by the plant [80]. The major regulators of nodule development are phytohormones. Some of them play distinct or even opposite roles in the organogenesis of determinate and indeterminate nodules [81]. Plant hormones are also produced by rhizobia and these may too affect nodulation process [82].

**Fig 5:**
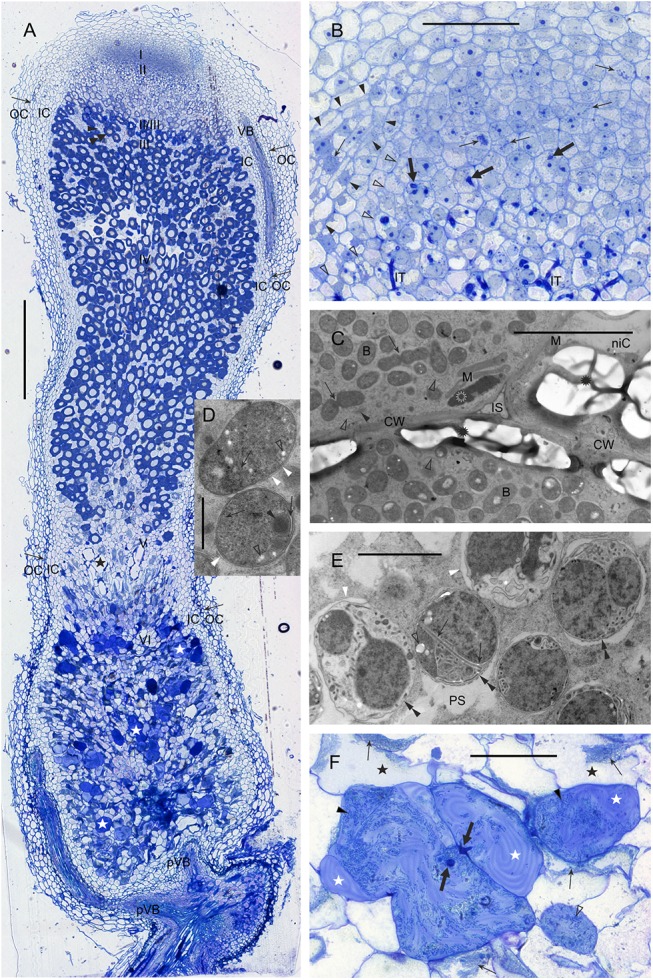
The structure of *A*. *glycyphyllos* root nodule. A. The general anatomy and ultrastructure of *A*. *glycyphyllos* root nodule. I–nodule meristem; II–differentiation zone of the bacteroid-containing tissue (= early symbiotic zone); II/III–starch-rich interzone II/III; III–nitrogen-fixing zone; IV–senescent zone; V—zone with infected cells degraded; VI—saprotrophic zone; IC–inner cortex of the nodule; thin arrow–the so-called nodule endodermis; OC—outer cortex; pVB—proximal vascular bundles; VB–vascular bundle; arrowhead—non-infected cells of the bacteroid-containing tissue; double arrowhead—infected cells of the bacteroid-containing tissue; black asterisk—degraded infected cells; white asterisk—cells populated by saprotrophic rhizobia embedded in a matrix. Bar = 500 μm. B. *A*. *glycyphyllos* nodule meristem and differentiation zone of the bacteroid-containing tissue. Thin arrows—mitotic cells; thick arrows—the most distant infection threads visible in section; IT—infection threads, arrowheads—delimitation of a meristematic apex of vascular bundle; open arrowheads—lateral delimitation of the differentiating bacteroid-containing tissue (note that in this region the inner cortex is only two cells wide). Bar = 50 μm. C. The cell ultrastructure at the boundary of differentiation zone and the interzone II/III of the bacteroid-containing tissue of *A*. *glycyphyllos* root nodule. Open rosette—plastid (note its close association with mitochondria); rosette—starch grains; slim arrows—dividing bacteroids; arrowhead—dictyosome; open arrowheads—rough endoplasmic reticulum cisterns; M—mitochondria; niC—non-infected cell; IS—intercellular space; CW—cell wall; B–bacteroids. Bar = 5 μm. D. The symbiosome ultrastructure in the interzone II/III cell of *A*. *glycyphyllos* root nodule. Slim arrows—invaginations of bacteroid's cytoplasmic membrane; arrowhead—fine-granular inclusion; open arrowheads—poly-beta-hydroxybutyrate granules; white arrowheads—peribacteroid membrane. Bar = 1 μm. E. The symbiosome ultrastructure in the senescent cell of the bacteroid-containing tissue of *A*. *glycyphyllos* root nodule. Slim arrows—invaginations of bacteroid's cytoplasmic membrane; arrowhead—fine-granular inclusion; open arrowhead—poly-beta-hydroxybutyrate granule; white arrowheads—peribacteroid membrane; double arrowheads—bacteroid's cell wall; PS—peribacteroid space. Bar = 2 μm. F. The saprotrophic zone of the bacteroid-containing tissue of *A*. *glycyphyllos* root nodule. Black asterisks—degraded infected cells; thin arrows—compressed remnants of degraded bacteroids; white asterisks—matrix in degraded cells populated by of saprotrophic rhizobia; thick arrows—infection threads; arrowheads—colonies of saprotrophic rhizobia. Bar = 50 μm.

The rhizobia studied here, induced cylindrical nodules on the *A*. *glycyphyllos* roots, resembling the indeterminate nodules of such fabaceans as, e.g.; *M*. *sativa* [83], *T*. *repens* [84], *C*. *arietinum* [85], *B*. *pelecinus* [77]. Anatomically, the liquorice milkvetch nodules were differentiated into persistent apical meristem, bacteroid tissue consisting of infected and uninfected cells, as well as multi-layered nodule cortex, with a vascular system (Fig 5A). Within the bacteroid tissue, a developmental zonation was discernible (Fig 5A). Proximal to nodule meristem, cells were penetrated by infection threads (Fig 5B) and infected endocytotically by rhizobia, which was a symptom of the onset of bacteroid tissue differentiation. In the infected cells, differentiation involved considerable cell growth, symbiosome multiplication, proliferation of membranous organelles and the decrease of vacuolation. Gradually, the amyloplasts were formed and together with closely-associated mitochondria they were translocated into the vicinity of intercellular spaces. Dividing symbiosomes were observed, even in the cells adjoining the so-called interzone II/III (Fig 5C).

In the final stage of infected cell differentiation, which took place in the interzone II/III, starch grains increased the size rapidly and acquired an elongated form (Fig 5C), the single vacuole, located centrally, was formed close to the cell nucleus. In the symbiosomes, peribacteroid space was narrow (Fig 5D). Bacteroids were rounded or slightly elongated in sections, with both outer and cytoplasmic membranes (the latter forming occasional invaginations) clearly discernible. In fully differentiated infected cells, starch grains were smaller than in the interzone II/III cells and the symbiosomes were similar to those in the interzone II/III.

Gradually, the senescence zone was formed due to the degenerative changes in the ultrastructure of symbiosomes (Fig 5E). The peribacteroid space widened locally and bacteroids' cytoplasm became heterogeneous (Fig 5E). Invaginations of the cytoplasmic membrane gradually increased and took the shape of intricately twisted tubules. Concurrently, the degenerative changes occurred in the host organelles and tonoplast became fragmented (not shown). Infection threads with intact rhizobial cells were visible in the senescent zone. Within the completely degraded cells, only the so-called “ghost membranes” (remnants of bacteroids' cell wall) along with infection threads were present.

At the proximal end of the bacteroid tissue, closest to the point of attachment to the "parent" root, a large saprotrophic zone was formed (Fig 5A). After the infected cell’s degradation was complete, some of the dead cells became populated by a large number of rod-shaped rhizobia. They were embedded in a fibrillar matrix, which had a characteristic pinwheel pattern (Fig 5F) resulting from the orderly arrangement of fibrils.

The bacteroid tissue of nodule was surrounded by lateral tissues: a) the multilayered inner cortex with vascular bundles, b) the monolayered cortical endodermis, built of tightly-arranged cells and c) an outer cortex of loose parenchyma (Fig 5A).

Generally, the anatomy of *A*. *glycyphyllos* nodules was similar to that of other indeterminate ones [46, 77, 83–85], however, some traits specific to liquorice milkvetch nodules were observed at the ultrastructure level; e.g. the extensive invaginations of bacteroids cytoplasmic membrane, especially evident in the senescent bacteroids.

In summary, the six *A*. *glycyphyllos* nodulators studied, closely related to members of the *M*. *amorphae* and *M*. *septentrionale* species (AG17 and AG22) and *M*. *ciceri* bacteria (AG1, AG7, AG15 and AG27), harbor highly conserved *nodACH* and *nifH* genes and all these bacteria were clustered together in a clearly separate, strongly supported clade, in corresponding genes trees. The most plausible explanation for incongruence between phylogenies based on 16S rRNA and symbiosis genes, seems to be the lateral transfer of symbiotic information, from a common ancestor to the bacteria studied. Rhizobia specific to *A*. *glycyphyllos* have a narrow host range and they were classified to the new symbiovar “*glycyphyllae*”, based on phylogenetic analysis of the *nodA* and *nodC* genes. Nodules induced on liquorice milkvetch roots are typically indeterminate, in terms of their histological structure.
